# Description of *Phradonoma blabolili* sp. n. (Coleoptera, Dermestidae, Megatominae), with notes on the dermestid beetles from Angola

**DOI:** 10.3897/zookeys.293.4891

**Published:** 2013-04-19

**Authors:** Jiří Háva, Tomáš Lackner, Jana Mazancová

**Affiliations:** 1 Department of Forest Protection and Entomology, Faculty of Forestry and Wood Sciences, Czech University of Life Sciences, Kamýcká 129, CZ-165 21, Prague 6 - Suchdol, Czech Republic; 2 Institute for Tropics and Subtropics, Czech University of Life Sciences, Kamýcká 129, CZ-165 21, Prague 6 - Suchdol, Czech Republic

**Keywords:** Taxonomy, new species, Coleoptera, Dermestidae, *Phradonoma*, Angola

## Abstract

*Phradonoma blabolili* **sp. n.** from Angola is described and illustrated. Key to the Afrotropical “*Phradonoma nobile* species group” to which the newly described species belongs, as well as key to genera of dermestid beetles occurring in Angola is given. List of all species of Dermestidae known to occur in Angola hitherto is provided.

## Introduction

The family Dermestidae (Coleoptera: Bostrichoidea) contains about 1440 species and subspecies worldwide (Háva 2003a, Háva and Solervicens 2012). Its members are “mainly scavengers on dried proteinaceous material and are of economic importance because the family includes species that are pests of stored products or natural-history enemies” (Lawrence and Ślipiński 2005). Despite their species richness, only 14 species have been reported from Angola hitherto (Erichson 1843, Ferreira 1965, Háva 2003a, b, Kadej 2006, 2010, Mroczkowski 1968, Pacavira et al. 2006, Pic 1931, 1937). This doubtlessly small number is perhaps largely due to the 27 years of Angolan civil war (1975–2002) which was a serious impediment to entomological research; the actual number of species is undoubtedly much higher. After the end of the conflict, and especially in the recent years, specialists carrying out entomological research seem to be returning to Angola. The genus *Phradonoma* Jacquelin du Val, 1859 is distributed largely in Palaearctic and Afrotropical regions and one species has been introduced into the U.S.A. In this contribution to the taxonomy of Angolan Dermestidae (Coleoptera) we describe one new species of *Phradonoma* and provide a summary of the dermestid taxa occurring there.

## Material and methods

The type specimen of this new species has been collected using flight intercept trap in open savannah near Catabola, in the central Angolan province of Bié, in altitude 1300 m. The FIT trap has been placed near a small pond, and cow dung, rotting bananas as well as rotting fish were all used to attract insects. The attractants were placed in small plastic containers around the trap. When removing the male terminalia from the specimen, the entire abdomen was first severed from the rest of the body, subsequently macerated in KOH heated up to 90°C for a short while, cleared in 96% ethanol and thence the male genitalia was removed from the cleared abdomen. The habitus photographs of *Phradonoma blabolili* was taken by macroscope Leica 216 APO. The dissected male genitalia was macerated in 10 % solution of KOH heated up to 90 °C for a few minutes, cleared in Xylene and transferred into glycerin in small glass dish where it was observed. The photograph of the male genitalia has been taken with Olympus BX 41 camera. The map on Fig. 5 depicting the type locality of *Phradonoma blabolili* was downloaded from the Internet and subsequently re-drawn using Adobe Illustrator CS4. The type specimen is deposited in the collection of the senior author (JHAC).

Standard measurements have been made according to Háva (2006) and are as follows:

**BL** Body length - linear distance measured from anterior margin of pronotum to apex of elytra;

**BW** Body width - measured between two anterolateral humeral calli;

**PL** Pronotum length - measured from the top of the anterior margin to scutellum;

**PW** Pronotum width - measured between the two posterior angles of pronotum;

**EL** Elytral length- linear distance measured from shoulder to apex of elytron.

### Abbreviation

**JHAC** Private Entomological Laboratory & Collection, Únětice u Prahy, Prague-west, Czech Republic

## Results

### Subfamily Megatominae Leach, 1815 Tribe Megatomini Leach, 1815

#### Phradonoma blabolili sp. n.

urn:lsid:zoobank.org:act:93246F10-2B76-4573-9771-C6F3C84E7538

http://species-id.net/wiki/Phradonoma_blabolili

Figs 1 5

##### Type material.

Holotype, male, with printed label “Angola, Bié province, Catabola env., 15–27.11.2012, FIT trap, T. Lackner lgt.” (JHAC).

##### Description.

**Figure 1.**
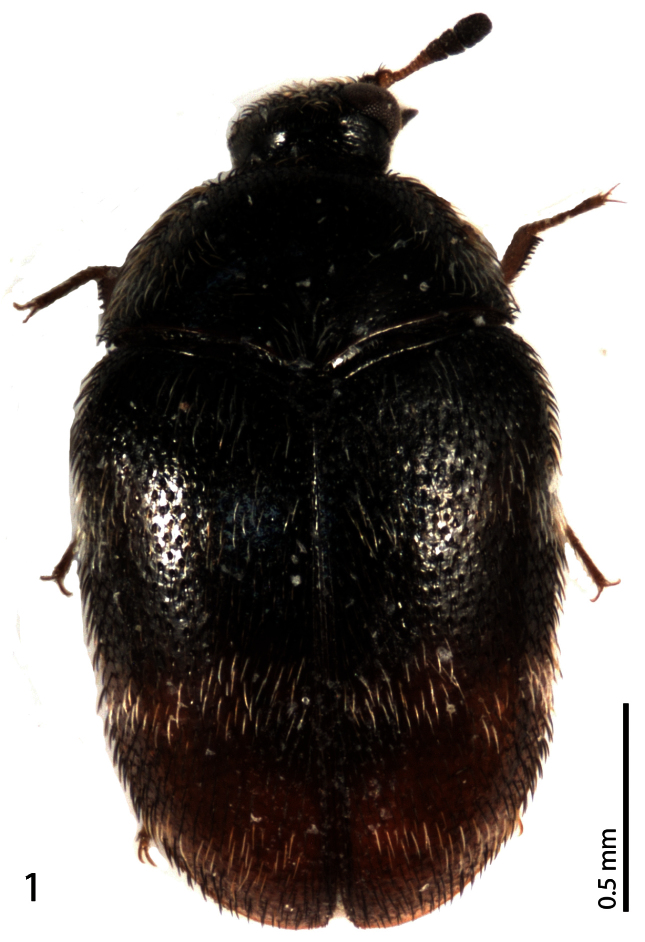
*Phradonoma blabolili* sp. n., habitus, dorsal view.

**Figure 2.**
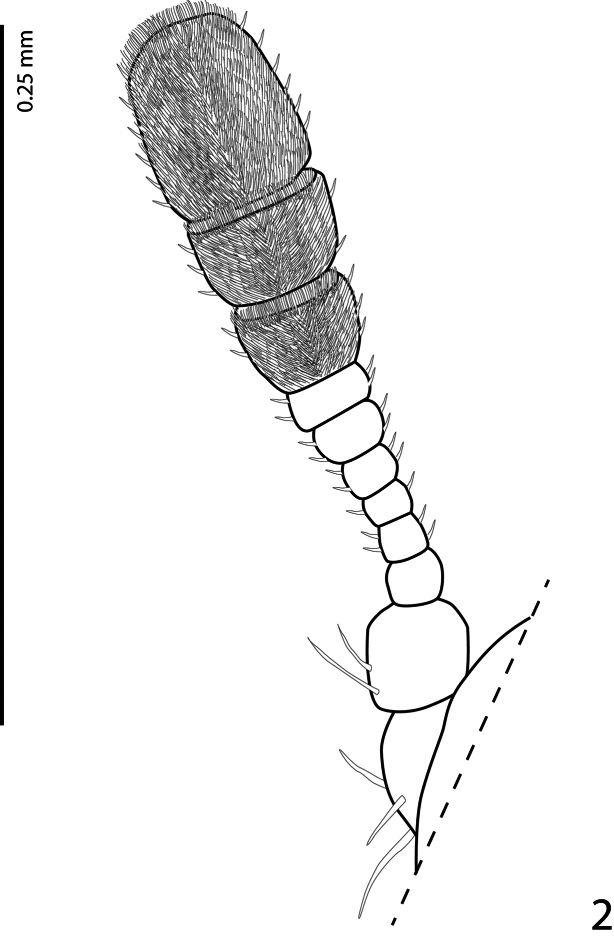
*Phradonoma blabolili* sp. n., antenna, dorsal view.

Male. Body measurements: BL 2.20 mm; BW 1.30 mm; PL 0.60 mm; PW 1.10 mm; EL 1.70 mm. Body (Fig. 1) dark brown and black, elongate oval. Head entirely black, coarsely punctuate, with decumbent light brown setae; maxillary palpi dark brown. Eyes large, with short microscopic setae. Antennae with 11 antennomeres, antennal club consisting of 3 antennomeres; antennomeres I-VIII brown, antennomeres IX-XI black, furnished with short setae (Fig. 2). Frons with small dark brown ocellus. Pronotum entirely black, shiny, sparsely and finely punctuate, with dark and semi-erect setae medially, white setae increase in number towards the lateral margins, posterior edges and in ante-scutellar area; lateral pronotal margins not visible from above. Scutellum small, black and triangular, asetose and impunctate.

Elytra black in anterior half, dark brown on posterior half, sparsely and coarsely punctuate; sparsely covered with semi-erect dark setae. Each elytron bears four transverse fasciae formed by intermixed white and yellow setae: the first situated near scutellum; second present anteriorly, reaching elytral suture; third fascia situated sub-medially reaching elytral suture; and the fourth fascia situated sub-apically, reaching elytral suture. Elytral epipleuron short, black, with dark setae.

Metaventrite finely punctuate with white, short, recumbent setae. Mesoventrite coarsely punctuate laterally, medially finely punctuate, and covered by white, short, recumbent setae.

All abdominal ventrites black, covered by short, white, recumbent setae; first abdominal ventrite with distinct oblique discal striae.

Legs. Tibiae and tarsi brown, femora anteriorly darkened and sparsely covered with fine white setae. Anterior tibiae with black spines along shaft.

**Figure 3.**
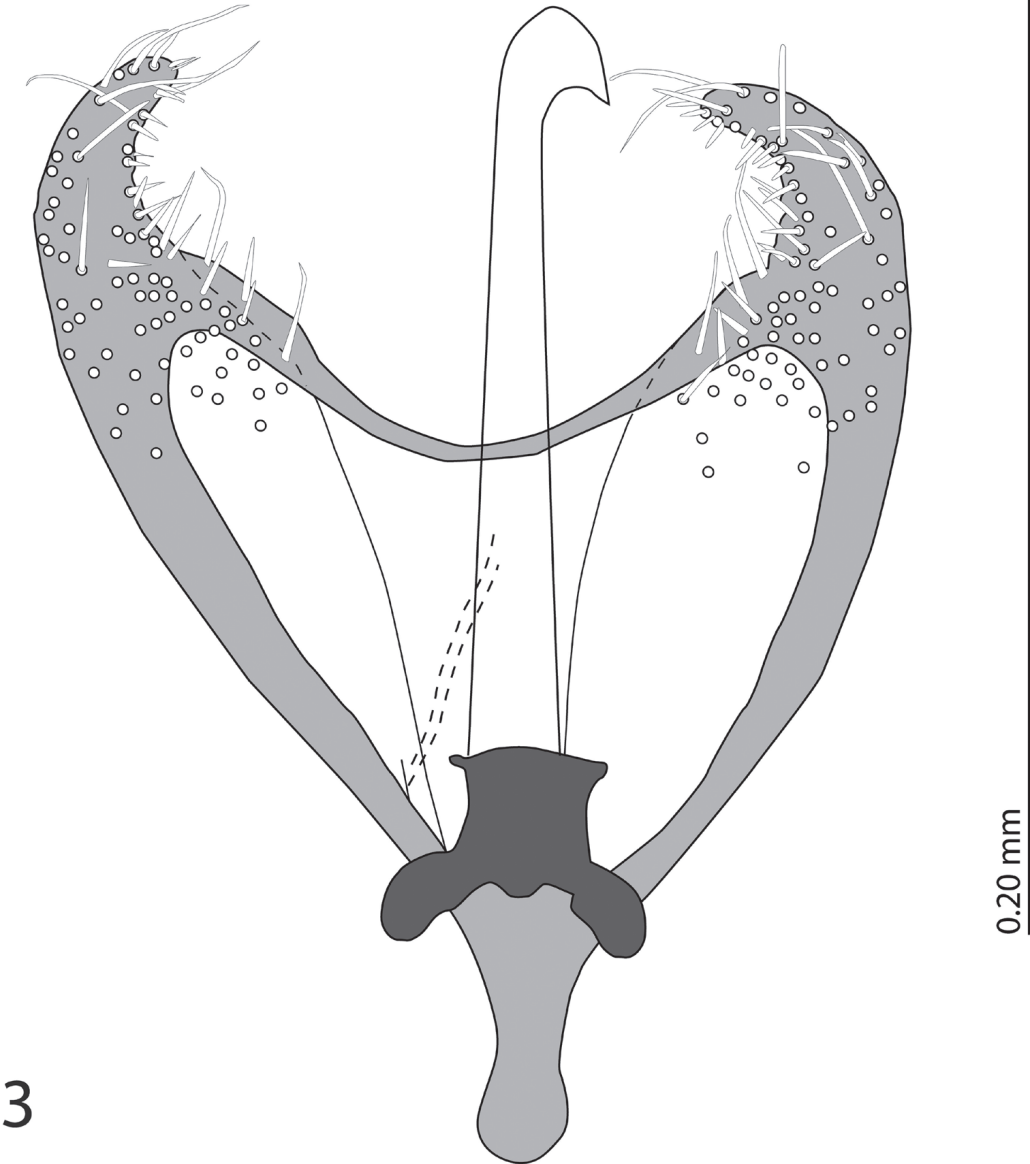
*Phradonoma blabolili* sp. n., aedeagus, dorsal view, showing the reconstructed penis.

**Figure 4.**
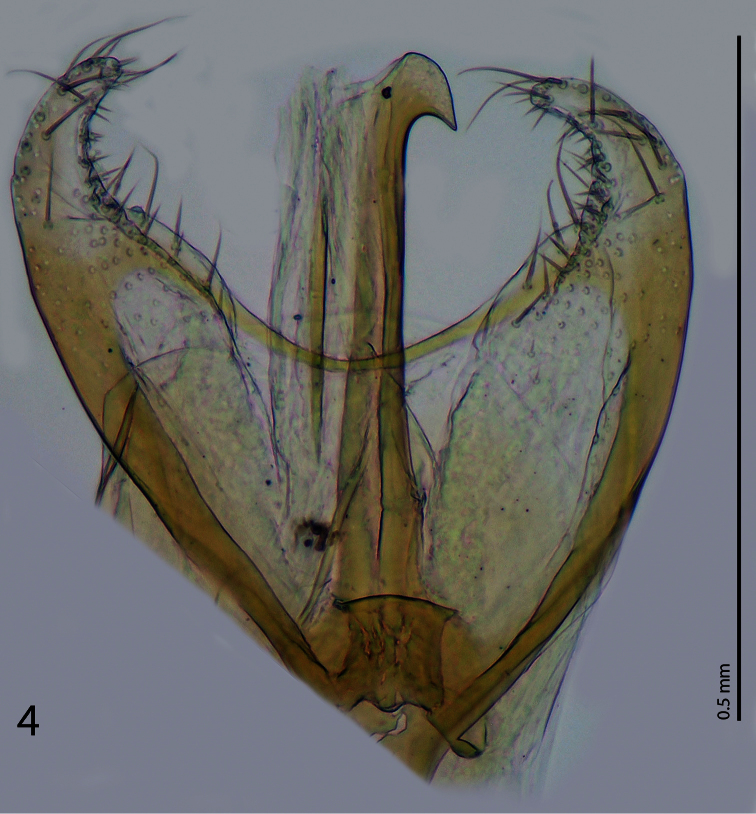
*Phradonoma blabolili* sp. n., aedeagus, depicting the damaged penis.

Male genitalia. Parameres widely ‘open’ connected anteriorly by a ‘bridge’, parameres apically with pseudopores and short setae; basal piece strongly sclerotized; penis apically with downward pointing ‘hook’. Penis has been slightly damaged during the manipulation with the aedeagus and therefore we decided to show the photograph as well as the drawing of the aedeagus depicting a reconstructed penis. (Figs 3-4).

Female unknown.

**Figure 5.**
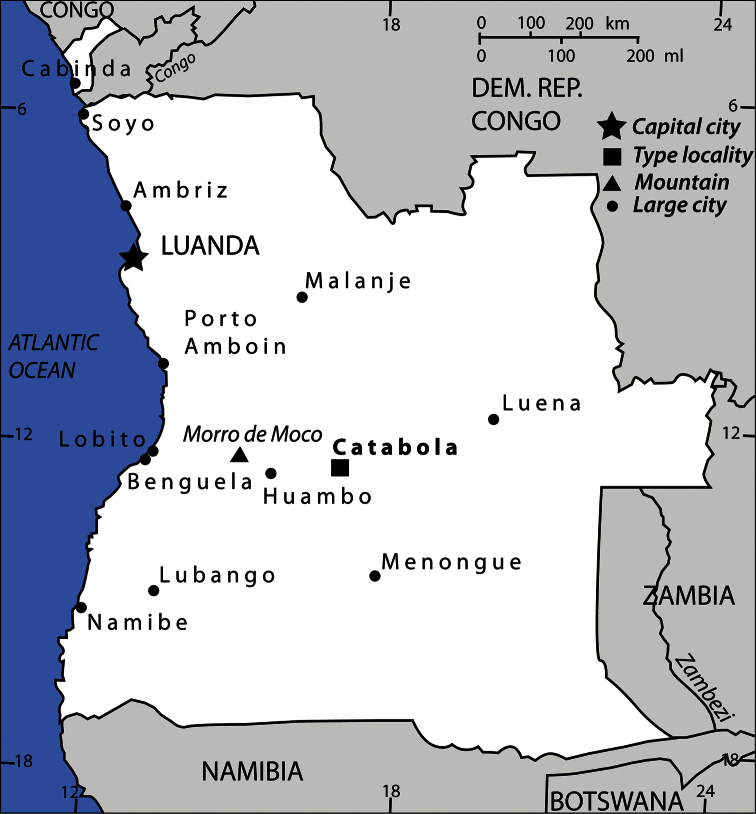
Map of Angola showing the type locality of *Phradonoma blabolili* sp. n.

##### Differential diagnosis.

This new species belongs to the genus *Phradonoma* Jacquelin du Val, 1859, and can be placed into the “*Phradonoma nobile* species group” (sensu Háva 2006; see also below). *Phradonoma blabolili* sp. n. is visually most similar to *Phradonoma cornelli* Háva & Hermann, 2009 and can be differentiated from it by the characters given in the key.

##### Distribution.

Known only from the vicinity of Catabola, Bié province, central Angola (Fig. 5).

##### Etymology.

Patronymic, dedicated to Martin Blabolil, (Kuito, Angola) who has been instrumental in providing all kinds of help during the visit of Tomáš Lackner in Angola.

### Key to the Afrotropical “*Phradonoma nobile* species group”

“*Phradnonoma nobile* species group” of the genus *Phradonoma* can be defined by the combination of the following putative synapomorphies: cuticle bicolored, dorsal body surface with bi- or tri-colored setae and antennal club consisting from three antennomeres (see also Háva 2006).

**Table:** 

1	Body form narrow, parallel, elytra without white setae, black with orange apex; antennal club with 5 antennomeres (Kenya, Namibia, Tanzania)	*Phradonoma babaulti* (Pic, 1921)
–	Body form oval, elytra with brown and white or grey setae, antennal club with 3 antennomeres	2
3	Berminal antennal antennomere triangular; elytra brownish-black except for three (sometimes only two) red, transverse bands and small circular spots; (Cyprus, England (intr.), Greece, Portugal, Spain, Algeria, Egypt, Eritrea, Libya, Morocco, Namibia, Nigeria, South Africa, South Sudan, Sudan, Tanzania, Tunisia, Zimbabwe, Afghanistan, „Caucasus“, India: Madhya Pradesh, Rajasthan, Uttar Pradesh, Iran, Iraq, Israel, Jordan, Pakistan, Qatar, Saudi Arabia, Syria, Tajikistan, Turkmenistan, United Arab Emirates, Uzbekistan, USA: Arizona (intr.))	*Phradonoma nobile* (Reitter, 1881)
–	Terminal antennal antennomere oval	4
4	Elytra with light fasciae of setae and apical spot	5
–	Elytra with isolated light spots of setae	7
5	Elytra with one orange transverse fasciae, small median orange patches and orange apical spot all covered by white setae; body length 2.30–2.70 mm; antennal club with 3 antennomeres (Botswana, Congo, Namibia, South Africa, Tanzania, Zambia, Zimbabwe)	*Phradonoma eximium* (Arrow, 1915)
–	Elytra dark brown or black and dark brown without median, orange or brown patches	6
6	Elytra dark brown, each elytron covered by slightly erected dark setae with three or four fasciae and small apical spot from light brown and white setae; body length 2.10–2.60 mm; antennal club with 3 antennomeres (Cameroon)*Phradonoma cornelli* Háva & Herrmann, 2009
–	Elytra black in anterior half, dark brown posteriorly, each elytron with four distinct transverse fasciae from grey setae; body length 2.20 mm; antennal club with 3 antennomeres (Angola: Bié province)	*Phradonoma blabolili* sp. n.
7	Elytra with isolated light spots of setae	8
8	Elytra black, without red, orange or brown parts. Body length 2.60–2.70 mm; antennal club with 3 antennomeres; each elytron with very small isolated 13–14 white spots (Kenya, Madagascar)	*Phradonoma albonotatum* (Pic, 1927)
–	Elytra with red, orange or brown parts	9
9	Pronotum with 5 isolated white patches, two in lateral parts, two medially and one near scutellum; body length 2.30–3.30 mm; antennal club with 3 antennomeres; elytra black with orange-brown apical part and with small white spots (Botswana, South Africa)	*Phradonoma borowieci* Háva & Kadej, 2006
–	Pronotum with two lateral white patches	10
10	Elytra near scutellum coarsely punctured with small humeral bump; body length 2.80 mm; antennal club with 3 antennomeres; elytra black, each elytron with 12 small, distinct spots of white setae on three or four very blurred fasciae and an apical spot (Cameroon)	*Phradonoma angelusi* Háva & Herrmann, 2009
–	Elytra near scutellum finely punctured with very large humeral bump; body length 2.40–3.20 mm; antennal club with 3 antennomeres; elytra black with orange apex, each elytron intermixed in brown setae with small patches of white setae (Namibia)	*Phradonoma namibicum* Háva, 2005

### Key to genera of Dermestidae hitherto known to occur in Angola

**Table:** 

1	Head without frontal ocellus	subfamily Dermestinae, genus *Dermestes*, 2
–	head with frontal ocellus	3
2	visible abdominal sternites with white and black pubescence	subgenus *Dermestinus* Zhantiev, 1967
–	visible abdominal sternites with concolorous pubescence	subgenus *Dermestes* Linnaeus, 1758
3	prosternum not forming a “collar”; mouthparts free	subfamily Attageninae, genus *Attagenus* Latreille, 1802
–	prosternum forming a “collar” under which mouthparts fit when the head is retracted	subfamily Megatominae, 4
4	dorsal and ventral surfaces covered by flat scales	genus *Anthrenus*, 5
–	dorsal and ventral surfaces covered by pubescence	7
5	antenna with 11 antennomeres	6
–	antenna with 10 antennomeres	subgenus *Anthrenodes* Chobaut, 1898
6	eyes with median margin broadly and deeply emarginate at about anterior 1/3	subgenus *Anthrenus* Geoffroy, 1762
–	eyes with median margins complete	subgenus *Nathrenus* Casey, 1900
7	anterior tibiae with spines along shaft; antennal club with 3 antennomeres	genus *Phradonoma* Jacquelin du Val, 1859
–	anterior tibiae without spines	8
8	antennal club with 2 antennomeres, terminal antennomere of male big, flat and slightly vaulted	genus *Orphinus* Motschulsky, 1858
–	antennal club with 3-8 antennomeres	genus *Trogoderma* Dejean, 1821

### List of the dermestid beetles reported from Angola so far:

Subfamily Dermestinae Latreille, 1804

Tribe Dermestini Latreille, 1804

*Dermestes (Dermestes) ater* DeGeer, 1774

= *Dermestes cadaverinus* Fabricius, 1775

= *Dermestes domesticus* Germar, 1824

= *Dermestes cinereus* Motschulsky, 1848

*Dermestes (Dermestes) lardarius* Linnaeus, 1758

*Dermestes (Dermestinus) maculatus* DeGeer, 1774

= *Dermestes vulpinus* Fabricius, 1781

= *Dermestes senex* Germar, 1824

= *Dermestes lupinus* Eschscholtz in Mannerheim, 1843

Subfamily Attageninae Laporte, 1840

Tribe Attagenini Laporte, 1840

*Attagenus donckieri* Pic, 1916

*Attagenus fasciatus* (Thunberg, 1795)

= *Anthrenus gloriosae* Fabricius, 1798

*Attagenus hargreavesi* Pic, 1935

*Attagenus havai* Kadej, 2006

*Attagenus vestitus* Klug, 1855

= *Attagenus rhodesianus* Pic, 1927

Subfamily Megatominae Leach, 1815

Tribe Anthrenini Gistel, 1848

*Anthrenus (Anthrenodes) endroedyi* Háva, 2003b

*Anthrenus (Anthrenus) flavipes flavipes* LeConte, 1854

*Anthrenus (Nathrenus) maltzi* Kadej, 2010

*Anthrenus (Nathrenus) verbasci* (Linnaeus, 1767)

Tribe Megatomini Leach, 1815

*Orphinus (Orphinus) aethiops* Arrow, 1915

*Orphinus (Orphinus) incognitus* Háva, 2003b

*Phradonoma blabolili* sp. n.

*Trogoderma granarium* Everts, 1898

## Supplementary Material

XML Treatment for Phradonoma blabolili
